# The logics of metabolic regulation in bacteria challenges biosensor-based metabolic engineering

**DOI:** 10.15698/mic2018.01.610

**Published:** 2017-12-11

**Authors:** Matthieu Jules

**Affiliations:** 1 Micalis Institute, INRA, AgroParisTech, Université Paris-Saclay, 78350 Jouy-en-Josas, France.

**Keywords:** pyruvate transport, Bacillus subtilis, catabolite repression, two-component systems, malate, synthetic Biology, biosensor, metabolic engineering

## Abstract

Synthetic Biology (SB) aims at the rational design and engineering of novel biological functions and systems. By facilitating the engineering of living organisms, SB promises to facilitate the development of many new applications for health, biomanufacturing, and the environment. Over the last decade, SB promoted the construction of libraries of components enabling the fine-tuning of genetic circuits expression and the development of novel genome engineering methodologies for many organisms of interest. SB thus opened new perspectives in the field of metabolic engineering, which was until then mainly limited to (over)producing naturally synthesized metabolic compounds. To engineer efficient cell factories, it is key to precisely reroute cellular resources from the central carbon metabolism (CCM) to the synthetic circuitry. This task is however difficult as there is still significant lack of knowledge regarding both the function of several metabolic components and the regulation of the CCM fluxes for many industrially important bacteria. Pyruvate is a pivotal metabolite at the heart of the CCM and a key precursor for the synthesis of several commodity compounds and fine chemicals. Numerous bacterial species can also use it as a carbon source when present in the environment but bacterial, pyruvate-specific uptake systems were to be discovered. This is an issue for metabolic engineering as one can imagine to make use of pyruvate transport systems to replenish synthetic metabolic pathways towards the synthesis of chemicals of interest. Here we describe a recent study (MBio 8(5): e00976-17), which identified and characterized a pyruvate transport system in the Gram-positive (G^+ve^) bacterium *Bacillus subtilis*, a well-established biotechnological workhorse for the production of enzymes, fine chemicals and antibiotics. This study also revealed that the activity of the two-component system (TCS) responsible for its induction is retro-inhibited by the level of pyruvate influx. Following up on the open question which is whether this retro-inhibition is a generic mechanism for TCSs, we will discuss the implications in metabolic engineering.

The central carbon metabolism provides energy and biomass precursors to the entire cellular network. Its fine regulation is therefore essential to ensure optimal use of carbon sources and proper allocation of resources to the metabolic pathways. Most living organisms including bacteria can use various compounds as carbon sources, where these can be either co-metabolized or selectively used. The later phenomenon was first described by Jacques Monod, who observed a glucose-over-lactose preference for the Gram negative (G^-ve^) bacterium, *Escherichia coli*. Subsequent mechanistic studies in bacteria showed that preferred carbon source(s) (most often glucose) are able to restrain transport and catabolism of alternative carbon sources. This regulation, called carbon catabolic repression (CCR), imposes a strict hierarchy in the use of carbon sources and allows for optimal adaptation (*i.e.* fitness and growth rate) to environmental conditions. Usually, CCR involves transcriptional regulation to prevent transcription of catabolic genes and operons (catabolite represssion *sensu stricto*) and post-transcriptional regulation to prevent uptake or formation of the specific inducers of catabolic operons (inducer exclusion). In most G^+ve^ bacteria and in particular in *B. subtilis*, the glucose-mediated CCR operates at the transcriptional level via the master regulator of carbon metabolism CcpA. Repression of several targets, among which are the genes encoding the transporters of alternative carbon sources, then occurs upon binding of CcpA to regions in promoters called catabolite responsive elements (*cre* sites). Hence, CcpA can regulate up to 10% of the bacterial genome. While organic acids are considered to be low in the hierarchy of the CCR, malate is an exception in *B. subtilis*. Malate is rapidly metabolized by the cell, can be co-metabolized with glucose, and represses the use of alternative substrates by hijacking the usual glucose-mediated CcpA-dependent CCR. In addition to regulation by the CCR, uptake systems are usually induced in the presence of the metabolite they transport. This creates an additional level of regulation that avoids wasting resources by producing unnecessary carriers. It is a fundamental principle of design that can be understood in biology as the result of the optimization of fitness. In bacteria, signal transduction mechanisms, known as two-component regulatory systems (TCS), represent the major signal response cascade to environmental signals. TCSs are found in a wide variety of processes such as virulence, mobility, growth and metabolism. The canonical form of a TCS consists of a sensor kinase (SK) and a response regulator (RR). The SK detects an environmental signal and is capable of autophosphorylating from an ATP molecule to a histidine residue in its catalytic domain. The phosphate is then transferred to the aspartate of the RR, which often functions as a phosphorylation-dependent DNA-binding transcription factor (*i.e. *activator or repressor). In *B. subtilis*, there are at least thirty-four TCSs involved in regulating the central carbon metabolism and more particularly the entry of gluconeogenic substrates. Pyruvate is a small-molecule metabolite at the heart of the CCM and a gluconeogenic substrate assimilated by several bacterial species. Our initial results from a study to address the identification and characterization of pyruvate uptake and regulation in *B. subtilis *were recently reported in mBio (MBio 8(5): e00976-17) and are summarized and expanded here.

## THE PftAB COMPLEX FROM *B. SUBTILIS* OPERATES AS A PYRUVATE-SPECIFIC FACLILITATED TRANSPORTER 

**Figure 1:**
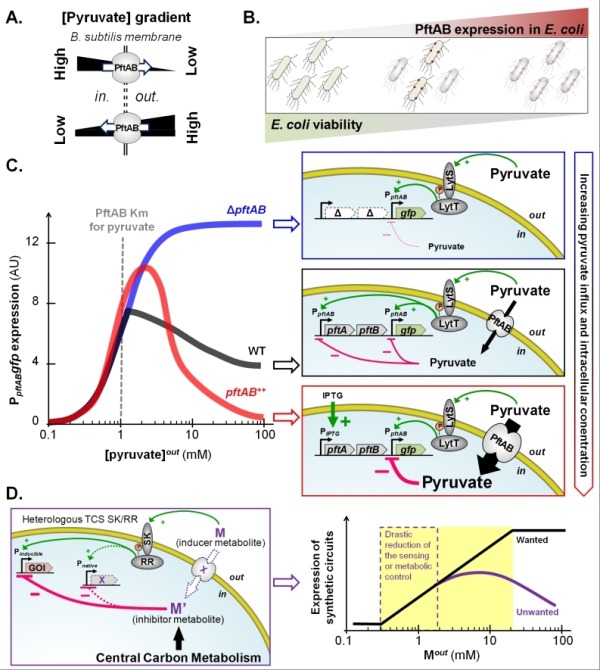
FIGURE 1: The bacterial pyruvate transport system PftAB and its complex regulation by the two-component system LytST. **(A) **The pyruvate facilitated transporter PftAB of *B. subtilis *can either import or export pyruvate depending on the concentration gradient of pyruvate across the cell membrane. **(B)** *E. coli *cell death or cell growth inhibition increases with the level of heterologous expression of *pftAB*. **(C) **Expression of *pftAB* in WT (grey), Δ*pftAB* mutant (blue), and *pftAB* over-expressing (red) *B. subtilis* cells grown in minimal medium with glutamate and succinate as carbon source, and pyruvate concentrations ranging from 0.1 to 100 mM (left panel). Extracellular pyruvate acts as the signal molecule for LytST, which induces expression of *pftAB*. However, when the pyruvate influx is high, LytST activity is drastically retro-inhibited. Consistently, in the Δ*pftAB* mutant, the level of induction is maximal as there is no influx of pyruvate (right panel). **(D)** In metabolic engineering, the expression of one (or more) gene(s) of interest (GOI) is (are) under the control of promoter(s) that can be activated by the use of inducer metabolite(s) (M). The activity of a heterologously expressed TCS may be retro-inhibited by the inducer or derivative metabolites (M or M') if naturally present in the host cell (left panel). As a result, the TCS-induced expression of a synthetic circuit will not exhibit a log-linear dose response as M increases. The distortion between the expected (wanted) and effective (unwanted) induction challenges the rational design of novel nature-inspired sensors (right panel).

We knew from previous genome-wide studies in *B. subtilis *that only the two operonic genes, *pftA* and* pftB* (renamed as such for *Pyruvate facilitated transporter*), whose products are predicted to be membrane proteins, are specifically induced in the presence of pyruvate. This prompted us to question whether PftA and PftB were involved in extracellular pyruvate utilization. As expected, deletion of either *pftA* or *pftB* drastically reduces growth on pyruvate as sole carbon source. We also learned from various purification and co-purification experiments that PftA and PftB form a hetero-oligomeric membrane protein complex. The next step was to show that the PftAB membrane complex effectively codes for a pyruvate transport system. During growth on pyruvate of the G^+ve^ bacterium, *Corynebacterium glutamicum*, pyruvate is taken up by the monocarboxylate transporter MctC, and as a result, a *mctC* mutant is unable to grow on pyruvate as the sole carbon source. When expressed in *C. glutamicum*, the *pftAB *operon functionally replaces *mctC* and enables pyruvate uptake and cell growth. As the gradient of pyruvate drove the PftAB-mediated transport of pyruvate across the cell membrane, we concluded that PftAB operates as a pyruvate-specific facilitated transporter (V_Max_~10.0 mmol.h^-1^.g of cells^-1^, Km^pyruvate^~0.1 mmol.L^-1^). Hence, PftAB is able to either import or export pyruvate in response to environmental changes in pyruvate (Figure 1A). What happens if PftAB is overproduced in a bacterial species where the intracellular pyruvate concentration is much higher than the Km of PftAB as it may lead to significant pyruvate outflow? Several lines of evidence suggest that the intracellular level of pyruvate in *E. coli* is at least 10 times higher than in *B. subtilis *(*i.e.* between 5 and 10 µM). Expression of *pftAB *under the control of inducible promoters in *E. coli* grown in rich medium revealed that the higher the level of expression of *pftAB*, the more toxic (unpublished data, Figure 1B). Although not yet demonstrated, it is reasonable to assume that PftAB overproduction in *E. coli* disrupts pyruvate homeostasis, and as a consequence leads to cell growth inhibition or cell death.

## INDUCTION, REPRESSION AND RETRO-REGULATION of *pftAB* EXPRESSION IN *B. SUBTILIS* ALLOW FOR OPTIMAL ADAPTION TO CHANGES IN ENVIRONMENTAL CONDITIONS 

We knew from early studies that the *lytST* operon, which codes for a putative TCS, is involved in the induction of *pftAB*. Combining various *in vitro* and *in vivo* experimental approaches, we learned that extracellular pyruvate acts as the signal molecule for the LytS SK, which in turn activates the LytT RR. Induction then occurs by binding of LytT onto two boxes upstream of *pftAB*. The promoter of *pftAB* also contains a putative *cre* site overlapping the -35 region. Consistently, both glucose and malate, the preferred carbon sources for *B. subtilis*, trigger the binding of CcpA upstream *pftAB* which results in its catabolite repression. However, an additional CcpA-independent mechanism represses *pftAB* in the presence of malate but not glucose. As an active malic enzyme replenishing the pyruvate pool is required for this repression, we hypothesized that the end product of the reaction (*i.e.* intracellular pyruvate) is responsible for this repression. Interestingly, a general principle of design of metabolic systems resides in feedback regulations that avoid metabolic systems to run out of control. The reader will find in the literature excellent reviews that cover the area of metabolic feedback regulations, especially where the end products are the signal molecules. Lesser known but not less important are feedback regulations where the inducer metabolite also mediates the feedback control. For instance, sugars such as fructose or sucrose induce expression of their own transporter but also activate the CcpA-dependent CCR to repress the use of alternative carbon sources. By doing so they partially repress their own assimilation. An attractive hypothesis was therefore that pyruvate is capable of feedback regulation

on LytST to avoid the metabolism to run out of control in the presence of high amount of pyruvate. We tested this hypothesis and although extracellular pyruvate triggers the induction of *pftAB*, this induction is strongly inhibited by the activity of PftAB (Δ*pftAB* versus *pftAB* overexpressing cells, Figure 1C). Hence, the higher is the influx of pyruvate, the stronger is the retro-inhibition of *pftAB* induction via LytST.

## IMPLICATION IN GENETIC ENGINEERING OF SYNTHETIC METABOLIC CIRCUITS AND OF BIOSENSORS 

Our unpublished data in *B. subtilis* suggest that the TCS retro-inhibition is not restricted to LytST. We are currently investigating the underlying molecular mechanism of these retro-inhibitions and whether these are widespread in the bacterial kingdom. This is an important issue in metabolic engineering as the induction of synthetic circuit expression by heterologous TCSs may be retro-inhibited by the cognate inducer metabolite if naturally present at high concentration in the host cell (Figure 1D). In addition, unless the inducer metabolite is not taken up by the cell (as for instance in the Δ*pftAB* mutant, Figure 1C), the activator activity of a TCS may be reduced by the inducer metabolite influx. This regulation also complicates the development of biosensors for health or for environmental bioremediation. Such biosensors are often inspired from sensor proteins such as TCSs, and the distortion between the expected and effective induction levels challenges the rational design of novel nature-inspired sensors (Figure 1D).

